# Factors Affecting the Intention to Use Exoskeleton for exercise and the Moderating Effect of Frailty

**DOI:** 10.1093/geroni/igab046.2482

**Published:** 2021-12-17

**Authors:** Choi Bomi, Susanna Joo, Changmin Lee, Chang Oh Kim, Yun Mook Lim, Hey Jung Jun

**Affiliations:** 1 Yonsei University, Seoul, Seoul-t'ukpyolsi, Republic of Korea; 2 Yonsei University, Seodaemun-gu, Seoul-t'ukpyolsi, Republic of Korea; 3 Severance Hospital, Yonsei University College of Medicine, Seoul, Seoul-t'ukpyolsi, Republic of Korea

## Abstract

This study aims to find relevant factors influencing the intention to use exoskeletons for exercise (IEE) among older adults and to analyze the moderating effect of frailty. The sample of this study is 310 people (65 or older) without cognitive impairment who completed an online survey. The intention to use exoskeletons was measured with three questions from the Senior Technology Acceptance Model (STAM). Potential relevant factors comprise sociodemographic characteristics, physical and psychological health, exercise, attitude towards aging, and social relationship. Linear regression analyses showed that depressive symptoms, regular exercise, attitude towards aging, and social participation were significantly related to IEE. People with more depressive symptoms and a negative attitude towards aging are more likely to have a higher level of IEE. People who exercise regularly and actively participate in social activities showed a higher level of IEE. Subgroup analyses were performed based on the frailty status measured with Korean Groningen Frailty Indicator (K-GFI). Among people without frailty (N=177), regular exercise, and social participation were positively related to IEE. The number of chronic diseases and social participation was positively related to IEE among people with frailty (N=133). The results of this study implied that poor health conditions lead to an increased need for exoskeletons. The results of this study also suggested that exercise and social participation work as facilitating factors in the context of gerontechnology acceptance. Results of subgroup analyses suggested that influencing factors on IEE can vary depending on the physical functional status.

